# Anomaly Detection in Asset Degradation Process Using Variational Autoencoder and Explanations

**DOI:** 10.3390/s22010291

**Published:** 2021-12-31

**Authors:** Jakub Jakubowski, Przemysław Stanisz, Szymon Bobek, Grzegorz J. Nalepa

**Affiliations:** 1 Department of Applied Computer Science, AGH University of Science and Technology, 30-059 Krakow, Poland; 2 ArcelorMittal Poland, 31-752 Krakow, Poland; przemyslaw.stanisz@arcelormittal.com; 3 Jagiellonian Human-Centered Artificial Intelligence Laboratory (JAHCAI), Institute of Applied Computer Science, Jagiellonian University, 30-348 Krakow, Poland; grzegorz.j.nalepa@uj.edu.pl

**Keywords:** machine learning, deep learning, anomaly detection, hot rolling, explainable artificial intelligence

## Abstract

Development of predictive maintenance (PdM) solutions is one of the key aspects of Industry 4.0. In recent years, more attention has been paid to data-driven techniques, which use machine learning to monitor the health of an industrial asset. The major issue in the implementation of PdM models is a lack of good quality labelled data. In the paper we present how unsupervised learning using a variational autoencoder may be used to monitor the wear of rolls in a hot strip mill, a part of a steel-making site. As an additional benchmark we use a simulated turbofan engine data set provided by NASA. We also use explainability methods in order to understand the model’s predictions. The results show that the variational autoencoder slightly outperforms the base autoencoder architecture in anomaly detection tasks. However, its performance on the real use-case does not make it a production-ready solution for industry and should be a matter of further research. Furthermore, the information obtained from the explainability model can increase the reliability of the proposed artificial intelligence-based solution.

## 1. Introduction

Every industrial asset, whether it is a specialized device, vehicle, or machine in an industrial factory, must undergo maintenance actions to assure its safety and reliability. There exist several strategies for handling the maintenance process, the four main strategies that can be found in the literature are [1]:

- *corrective*—replacement of an asset after it fails, referred also as run-to-failure,
- *preventive*—replacement of an asset after a predefined period of time,
- *condition-based*—asset is continuously monitored and replaced after it gets out of normal working conditions,
- *predictive*—monitoring the condition of an asset with the use of advanced statistical methods like machine learning.

The strategies above are listed from the simplest, where the maintenance is done on the elements that failed, to the most complex ones where the advanced models are used for prediction of the asset’s health. Although the cost of implementing a maintenance strategy increases with complexity, more advanced strategies can bring benefits in terms of total maintenance costs. Nowadays, most of the companies use preventive maintenance strategies, as this is relatively easy to implement and allows for the minimization of the costs of serious failures and downtime. Predictive maintenance is not yet fully adopted in many industries due to the fact that (1) analyzing real-life data with machine learning algorithms requires a huge amount of work devoted to proper cleaning, transformation, and labelling, and (2) decisions made by AI algorithms lack justifications, which limits their adoption in areas where wrong decisions may results in substantial money loses.

Predictive maintenance has a close link to anomaly detection, as a degradation process of an asset may be observed with the changing value of the selected anomaly metric. When we observe more anomalous observations than normally, it may be an important indicator to undertake maintenance actions. However, to apply proper maintenance actions, the engineers must be aware what measurements led to the degradation, so they can find a root cause of the problem.

To address these problems, in this paper we propose a method that (1) uses unsupervised learning to limit the costs of data labelling and improve the robustness of the method, and (2) uses XAI to provide justifications of the decisions of the model to allow experts double check the AI decisions. The paper is an extension of our preliminary work published in [2]. The original contribution presented herein includes the design and evaluation of an unsupervised approach towards modeling the degradation of an industrial asset which uses variational autoencoder (VAE) as the learning algorithm. To explain the labels of the autoencoder, we build a surrogate model with Random Forests for VAE anomaly classification. The surrogate model is then explained with the use of the SHAP method to determine what measurements lead to anomalies according to our model. The explanation phase is model-agnostic and not tightly bounded with the anomaly detection technique, hence is robust enough to be applied to other algorithms. This approach shows superior results in comparison with our previous works and state-of the art anomaly detection methods. Moreover, we consider a real-life industrial use case from the steel industry, i.e., the steel hot rolling. The data was provided by ArcelorMittal Poland (AMP), and experts from the company co-developed and evaluated the resulting models.

The rest of the paper is organized as follows. In Section 2 we present a description of our real-life industrial case, which we later use as the evaluation case for the approach. We also used the turbofan engine dataset described in Section 3 for the sake of reproduction and benchmarking, as it is publicly available. In Section 4 we present state of the art in the area of predictive maintenance, with the main stress put on unsupervised and semi-supervised methods. In Section 5 we present our solution based on variational autoencoder and anomaly detection algorithms. In Section 6 we present results for both the benchmark case and our real life use case scenario. In Section 7 we summarize the work and provide discussion of the results.

## 2. Hot Rolling Process

Our work is dedicated to hot rolling, which is one of the steps in the steel manufacturing process. Currently, steel is most often produced in a continuous casting process in the form of slabs, billets or blooms. To give the steel required shape and dimensions, it must be rolled in very high temperatures. In this study we use data from Hot Rolling Mill (HRM), also referred as Hot Strip Mill (HSM), which is a part of the ArcelorMittal Poland company. The plant was built in 2007 and is one of the most modern rolling mills in Europe. In this plant the slabs, which are about 200 mm thick and 10 m long are transformed into flat plates which have 10–100 times lower thickness and longer length length. The industrial process is fully automated and well-measured, which allows for the production of high-quality products.

### 2.1. Process Description

**Figure 1:**
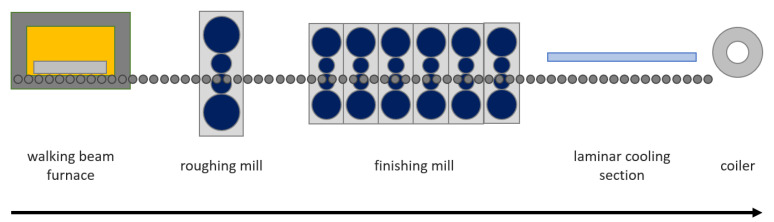
Simplified hot rolling mill process flow.

Figure 1 represents the most important elements of the hot rolling process. At first, a cold slab enters the walking beam furnace, where it is heated for several hours to reach the temperature of about 1200 C. After leaving the furnace slab it is transported by a conveyor belt to the roughing mill (RM). The RM consists of one pair of horizontal rolls and one pair of vertical rolls, which reduce the thickness and width of a slab. Every slab passes back and forth the RM many times, giving a total of five to nine passes. Before each pass, the slab is sprayed with pressurized water in a descaler to remove any scales from the surface of the steel. After the RM section, the transfer bar (which is the name of this intermediate product) is transported to the finishing mill (FM), which is the most complex section of the process. FM consists of six rolling stands in tandem that reduce the thickness of the steel to the desired value. As the rolling process progresses, the temperature of the material decreases to reach approximately 900 ${}^{\circ }$C at the last stand of the FM. After rolling, the steel is cooled to a predefined temperature in a laminar cooler, which uses water to lower the steel temperature. To produce high-quality steel process parameters (i.e., rolling force, looper tension, rolling speed), it must be precisely tuned and controlled to meet the product requirements. Except for the final dimensions, it is crucial to provide a product of desired mechanical (tensile strength, yield stress, elongation, grain orientation), visual (surface defects) and geometrical (flatness, wedge).

The proper maintenance of the line is also a key factor which affects the quality and reliability of the process. In HSM the manufacturing process is divided into campaigns where several dozens of slabs are treated. The length of the campaign is determined mostly by the wear of the work rolls, which have their roughness reduced due to contact with the steel strip. After each campaign, a short planned maintenance is done to replace the used elements (particularly work rolls) and perform minor maintenance actions. Additionally, in a predefined period of time, a longer stoppage is made to perform more complex maintenance.

### 2.2. Hot Rolling Dataset

A data set from the hot rolling mill consists of the average processing parameters for every slab/coil. For every observation, additionally to line measurements, we have the primary data input (PDI), which are features like weight, input and output thickness, input and output width, and chemical composition. As the whole process is very complex, we have focused our research only on the area of finishing mill. This includes features like rolling forces, looper tensions, temperatures, and rolling speed. The measurements related to this area together with PDI make a total of 32 distinct features.

The data set was cleaned from any outliers, but also limited to the most commonly rolled steel grades, to lower the total variance in the data set. Without such assumption, the model could assign rare products as anomalies, because its rolling recipe might be much different from common products. We also do not include the coils from the very beginning of rolling as they have much lower rolling speed, which could also falsify the predictions. We also filter out the products, which had some quality issues. With such assumptions, we have over 12,000 coils from three months of production in our data set.

**Figure 2:**
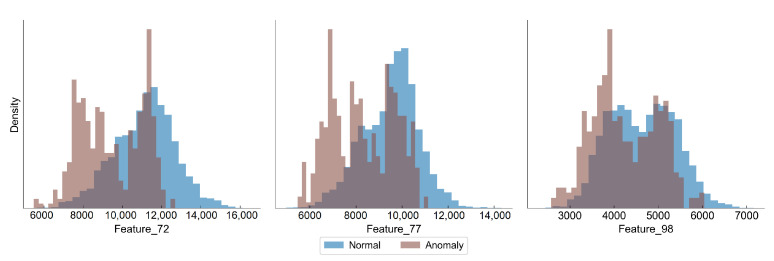
Distribution of features in normal and anomaly samples in hot rolling data set.

Figure 2 presents distributions of the selected features, in which we have observed a difference between the distribution of normal and anomaly (close to the campaign end) samples. In all features there is an observed shift towards lower values as the degradation progresses, although the overlapping of the distributions is significant.

**Figure 3:**
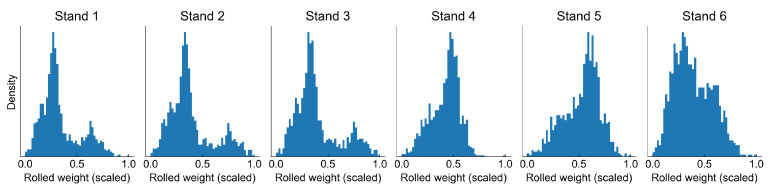
Histograms of work roll usage in stands of the finishing mill.

Each observation has a production time and production campaign number assigned, based on which we have calculated a cumulative sum of slab weights rolled at each step of the campaign. We use this value as our health indicator. However, it must be pointed out that we cannot calculate the remaining useful life (RUL) directly as the campaigns rarely end with failure. The rolling campaigns are planned in such a way that the asset does not exhibit too much abnormal behaviour, as this would definitely lead to quality issues, which obviously must be avoided. Normally rolls are replaced after each campaign; however, some of them can be used for two campaigns straight. The campaigns themselves can also be of various lengths, therefore the total roll usage may vary substantially. Figure 3 presents a scaled usage of work rolls on different stands.

Analysis of work roll usage distribution can bring some important observations. The first three stands have the same distribution, which means that these stands have the work rolls changed always at the same time. This is also true for stands 4 and 5. The distribution of the last stand is different from all other distributions, so it must be replaced at some other points in time, probably in some cases even during a campaign. This makes the analysis of the degradation process more complex, as the wear of work rolls may not be at the same level.

For this study, we selected coils with cumulative campaign weight above the 96th percentile of all the observations as the anomalous samples and between the 10th and 55th percentile as normal observations. The model is trained on normal observations; prediction metrics are calculated on the test set, which is used to optimize the model (hyperparameter tuning) and finally the model is scored on a separate evaluation data set to determine its performance.

## 3. Turbofan Engine Simulation

**Figure 4:**
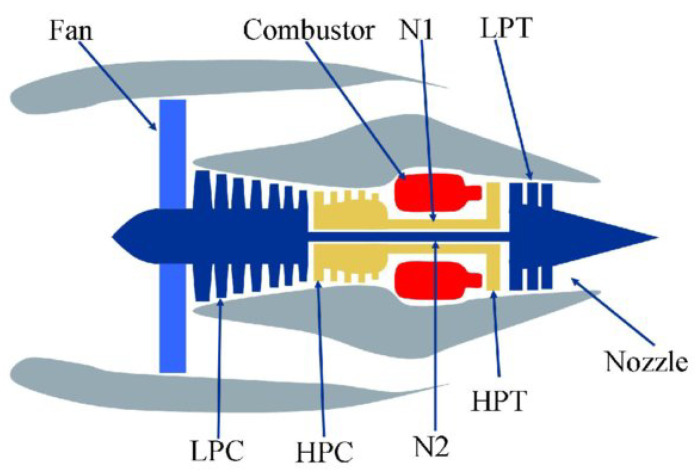
A schematic diagram showing the key components of turbofan engine, which is simulated in C-MAPSS [4].

The C-MAPSS data set [3] is one of the most widely used data sets for prognostics and health management research. It contains the results of a turbofan engine simulation using a C-MAPSS (Commercial Modular Aero-Propulsion System Simulation) software provided by NASA (National Aeronautics and Space Administration). Figure 4 shows a simplified diagram of the simulated system.

In the data set there are 3 operational parameters and 21 measurements of hundreds of turbofan units working under different conditions. The measurements include features such as zone temperature, zone pressure, fan speed, fuel flow, coolant flow, etc.

All units undergo a gradual deterioration of the high-pressure compressor (HPC), which leads to its failure. Each observation in the data is one cycle (flight) of a certain unit. The assumption is that the degradation rate depends on the number of factors and is different in each engine. Moreover, the initial wear of the engine might differ from unit to unit.

The data set is divided into four separate cases with different complexity. In the simplest scenario, all units operate under the same conditions and there is only one cause for the degradation. In the most complex scenario, the turbofan engine units are working under six different conditions and can exhibit two different reasons for failure. In this study, we have selected the FD004 data set, which is the most complex one.

**Figure 5:**
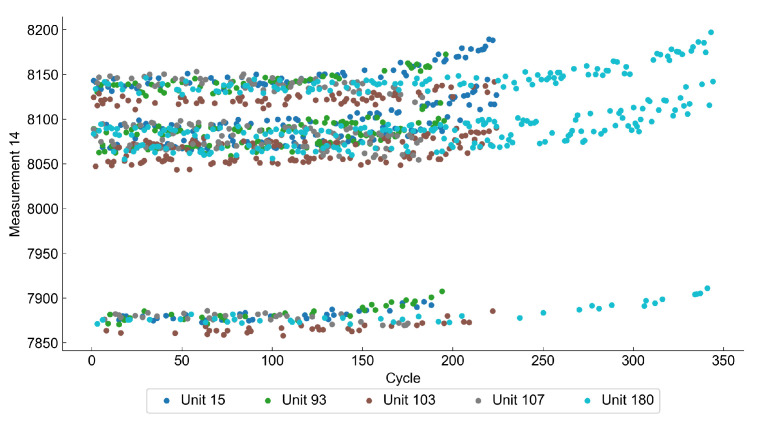
Plot of a single feature against cycle number of five randomly selected units in a C-MAPSS data set. The gradual deterioration of each unit is observed, starting at different cycles.

**Figure 6:**
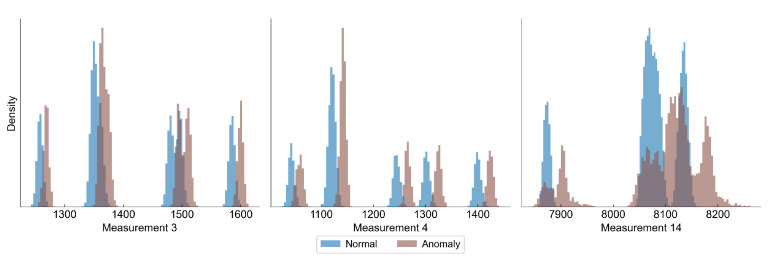
Histograms of three features from C-MAPSS, showing the distribution of normal samples (RUL above 130 cycles) and anomaly samples (RUL below 20 cycles). The measurements of anomaly samples exhibit a shift towards higher values.

Figure 5 shows how the value of a selected feature changes with the number of cycles. The increase of the value from a baseline may be considered as a sign of the anomaly and deterioration process. Figure 6 shows the distribution of some selected variables for normal and abnormal observations.

As the data consists of only run-to-failure cases, it can be easily used for the estimation of the machine’s remaining useful life, however it might as well be used in unsupervised methods where the value of the remaining useful life can be used not to train the model, but only to benchmark its performance. We must point out that the data is simulated and it can poorly reflect real life conditions, so these are the ones that should interest the researchers the most. However, it proved itself to be a good solution to evaluate the accuracy of different methods and architectures, which is why we have decided to use it for validating our method.

In the C-MAPSS dataset we limit the train set only to normal samples, which we defined as having RUL over 130 cycles. This value is commonly used in research made on this data set [5,6]. For the train and validation data sets we take the data with RUL above 130 cycles and below 20 cycles. We have set a 20 cycle limit as a threshold value for which we consider all observations to be abnormal. Therefore, the data set is labeled as normal if it has an RUL of 130 or more and as abnormal if it has an RUL below 20. The rest of the data is considered to be in an intermediate state, so it is not used for learning and evaluation.

## 4. Related Works

In this section, we present works in the research area that are relevant to the original contribution of this paper.

**Figure 7:**
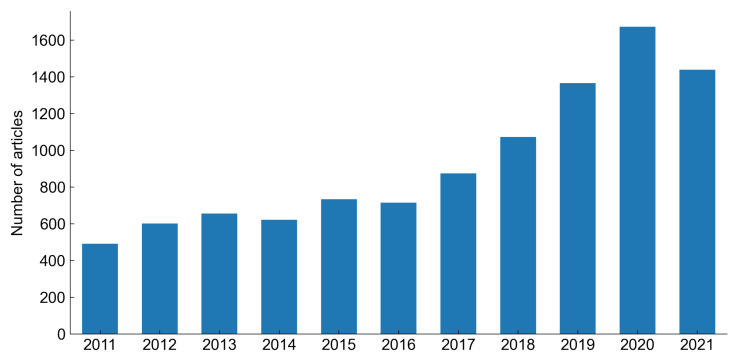
Number of articles with ’predictive maintenance’ keyword found in Scopus database by year of publish.

In recent years, there is an increasing number of articles related to predictive maintenance strategies. Figure 7 shows that the number of articles published yearly increased over 300% in the last 10 years. The increasing interest in predictive maintenance is connected with the so-called fourth industrial revolution and the development of the industrial Internet of Things technologies [7]. Raising self-awareness of the machine, which enhances its ability to self-diagnose and estimate its state of health, is one of the primary goals of cyber-physical systems (CPS) and Industry 4.0 [8]. The most effective solutions towards the development of predictive maintenance models are data-driven methods, which employ machine learning algorithms with a particular emphasis on deep learning methods [9].

Data-driven approaches for prognostics and health management (PHM), which are closely connected with predictive maintenance, use various methods like artificial neural networks (ANN), support vector machines (SVM), Bayesian methods, Markov models, regressive models (i.e., ARIMA), proportional hazards model [10], principal component analysis (PCA) T^2^ statistic, clustering methods [11] or anomaly detection. There is no single recipe for the development of machine learning algorithms for predictive maintenance. Depending on the nature of the problem and availability of the data, the learning process might be either:

- *supervised*—when we are in possession of a data indicating the asset’s health. An example of such data would be the machine’s remaining useful life (RUL), which is defined as “the length from current time to the end of the useful life” [12]. Such information can then be used as a target for our machine learning model.
- *unsupervised*—when we train the model without prior information about the machine’s state of health.
- *semi-supervised*—when we have some information about the condition of an asset, but not all data points are labeled or we do not use this information directly for training the model. An example of such an approach is when we have knowledge of previous failures, but we do not use it to train the model directly.

Although the first case is most convenient for researchers, it is rarely available in real life scenarios. Some of the many reasons are that in many cases machines exhibit a long-term degradation process which is difficult to capture and the fact that the asset rarely runs until its end of life [13] (due to undertaken preventive maintenance actions). To improve reliability of the models in the development of prognostic algorithms, it is reasonable to validate the model on a database containing well-documented information about failure. One such databank is provided by the NASA Prognostics Center of Excellence [14]. However the data in such repositories usually comes from laboratory experiments or simulations and thus may not always reflect the real production environment. Therefore, in real use cases semi-supervised and unsupervised methods may be more applicable, as they do not require extensive data labelling. However, these are much more difficult to evaluate, especially if there is no information about previous failures.

### 4.1. Anomaly Detection for Predictive Maintanance

Anomaly detection, also known as outlier detection, is a family of methods for recognizing observations which do not look like the majority of the data. The general idea behind anomaly detection is that the model learns on samples from normal working conditions and then tries to classify new observations based on their similarity to the learned scheme. Romotsoela et al. [15] reviewed some of the algorithms, which may be used for outlier detection. The list includes k-Nearest Neighbours (kNN) with local outlier factor (LOF) or connectivity-based outlier factor as a metric, one-class support vector machines (OCSVM), artificial neural networks and genetic algorithms. Other methods for the task of outlier detection include autoencoders (a specific type of artificial neural network), isolation forest [16] and clustering algorithms like k-Means and DBSCAN [17]. All listed anomaly detection techniques belong to the family of unsupervised learning models, as they do not use labeled data in the learning process. Although those methods require no data labelling, which can extend the range of applications to use cases where no labels are available, they create another issue that must be tackled by the researchers—it is necessary to set up parameters based on which a distinction between normal and abnormal observations is made. For instance, in kNN it is the number of neighbors, in OCSVM it is the gamma parameter, in isolation forest we need to determine the contamination parameter and in autoencoders (which make classification based on reconstruction loss) it is the threshold value.

Many researchers used machine learning for anomaly detection methods for predictive maintenance related tasks. Lindemann et al. [18] used k-Means clustering and LSTM Autoencoders for anomaly detection in a hydraulic press. Reder et al. [19] used k-Means clustering for fault detection in wind turbines. Renström et al. [20] also investigated anomalies in wind turbines, but they have used a deep autoencoder architecture for this purpose. Essien et al. [21] used a convolutional LSTM autoencoder for the detection of abnormal situations in a high-speed aluminium can-making machine.

The study on the degradation process of turbofan engine based on C-MAPSS data set is extensive and researchers used a variety of approaches. Da Costa et al. [22] used a Deep LSTM Adversial Network, Eleffsen et al. [23] combined Restricted Boltzmann Machine, LSTM and fully connected network, Khelif et al. [24] used Support Vector Regression, Mosallam et al. [25] used a Bayesian approaches. All listed articles uses the remaining useful time directly as the target variable of the model. Chen et al. [26] used a greedy kernel PCA to extract relevant features from the C-MAPSS dataset. Wu et al. [27] proposed a method which combined clustering and Hidden Markov Models. Al Batanieh et al. [28] used a deep autoencoder network to estimate the health of the machine by evaluating the reconstruction error of the model. This approach is the closest to the one we are proposing in this paper. Song et al. [29] also used an autoencoder architecture, however its task was only to reduce the dimension of data and it was later combined with a supervised learning method using a Bi-LSTM network.

In recent years, deep learning approaches using different kinds of autoencoders seem to gain more attention from researchers. When looking at the search result of the term “autoencoder predictive maintenance” in the Scopus database, there appears to be no paper from before 2016 and there were only 4 papers up to 2018. From 2019 to 2021, we have found 63 papers fitting this search term, which shows the increasing interest in this research area.

### 4.2. Autoencoders

**Figure 8:**
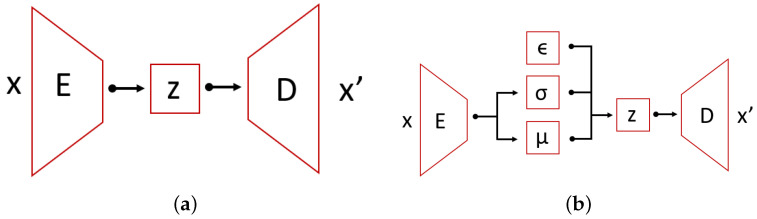
Schematic idea of autoencoder and variational autoencoder architecture. (**a**) AE; (**b**) VAE.

An Autoencoder (AE) [30] is one of many feed-forward neural network architectures. Autoencoders are gaining popularity recently in anomaly detection due to their flexibility. Their characteristic feature is having encoding layers followed by decoding multilayer neural network architecture connected through a latent multidimensional space (see Figure 8). During the iterative optimization (learning) process, the input is transferred to its output. The network tries to minimize the reconstruction error that propagates through the network. In this specific architecture, it is assumed that the latent space consists of a lower number of dimensions than the input data, which is also referred to as a bottleneck of the network. Therefore, we are dealing with the compression of the information followed by the decompression scheme. This can be very useful in terms of anomaly detection, because it can be assumed that we are only able to reconstruct properly the inputs similar to the ones that the network was trained on.

The main problem of applying autoencoders is the decision regarding the right dimension of the latent space. The change of this parameter influences the adjustment between fit of the data and model flexibility. For AE, we measure the anomaly through the performance of the reconstruction error [31]. The alternative is to analyze the latent space (i.e., indicate outliers with the isolation forest algorithm). However, this approach can bring some drawbacks (due to the nonlinear internal data structure), and therefore we need to derive a metric on latent space.

A Variational Autoencoder (VAE) [32] is a special case of an autoencoder which encodings’ distribution is regularized during the training. In other words, VAE add structure to the latent space. For a well-learned model, the multidimensional latent space is characterized by its internal structure. This information is used by the decoder part for the reconstruction of the input. In the same way as in AE, the VAE architecture is comprised of two main parts: an encoder and a decoder with an intermediate compressed low-dimensional layer space. What is characteristic to VAE is that the encoder maps the data to a posterior distribution. One of the common choices is to use univariate Gaussian distribution—this is post-variational inference which produces a family of candidates for variational inference. During the backpropagation, among families of solution latent space is organized by making the distribution returned by the encoder close to a standard normal distribution. The regularized term is described by the Kullback-Leibler divergence metric between the approximation and target. The overall solution can be found by gradient descent over all parameters. The total loss in VAE is defined as the sum of the reconstruction loss and the Kullback-Leibler divergence. Higgins et al. [33] have proposed to include a β coefficient, which regularizes the KL divergence term. The reconstruction loss is usually defined as the mean squared error between input and output, but other distance metrics might also be used. The formulae defining each term are as follows:

(1) $$R_{loss}=\frac{1}{n}\sum_{i=1}^{n}{\left(X_{i}-{\hat{X}}_{i}\right)}^{2}$$

(2) $$KL_{loss}=\sum_{i=1}^{n}\left(\mu_{i}^{2}+\sigma_{i}^{2}-\log\sigma_{i}-1\right)$$

(3) $$L=R_{loss}+\beta KL_{loss}$$

where $KL_{loss}$ is Kullback-Leibler divergence loss, $R_{loss}$ is reconstruction loss, *L* is total loss, $X_{i}$ is the ith input in a batch and ${\hat{X}}_{i}$ is the ith output in a batch, *n* is the number of batches, μ is the mean of latent space distribution, σ is the standard deviation of latent space distribution and β is a regularization term, which is model hyperparameter.

### 4.3. Explainable AI

Explainable artificial intelligence aims at bringing transparency, trust, and intelligibility to automated decision making systems [34]. This is especially important for models that are considered black-box mechanisms (e.g., Deep Neural Networks, Random Forests, Boosting, etc.) and are applied to areas where better understanding of an automated decision process is highly desired. In PM, explainable AI can be efficiently used for root cause analysis at the AI model level. It is worth noting that semantically there is a mismatch between the causality that human understands and the causality that the model uses. The former refers to the causality of real world mechanics, the latter to the simple correlation between input and output, which may not be the causation in reality. However, in industrial settings which depend highly on automated decision making, transparency in the decision process is as also important. In [35] the transmission light imaging was used for the analysis of machinery state to detect possible faults and implement maintenance upfront. Authors embedded the method with Bayesian linear regression models to achieve explainability. In [36] the authors used LIME and evaluated quality of explanations on various predictive maintenance datasets. An overview of XAI for predictive maintenance in the aerospace area was presented in [37]. In [38] QARMA algorithm was introduced for predictive maintenance in industrial IoT applications. Instead of using black-box model and the explainability wrapper, the authors build glass-box rule-mining model that is inherently interpretable. Although the area of XAI is extensively developed, there is still a need for research in the predictive maintenance field. In the following sections, we describe how high accuracy anomaly detection models can be combined with state of the art XAI methods to justify the detected anomalies.

## 5. Anomaly Detection Using Variational Autoencoder with Explanations

**Figure 9:**
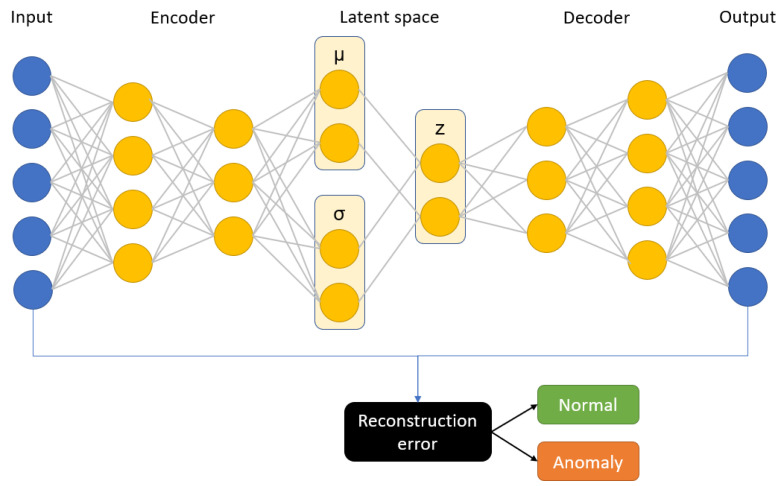
Schematic diagram of variational autoencoder.

In this study, we have proposed a solution for unsupervised anomaly detection in an asset where a deep variational autoencoder is learned on the normal process data and then it is used as a predictor of anomaly score. Based on the mean squared error between the original observation and reconstruction, we can classify the samples as either normal or anomalous. A threshold value for the classifier is determined during the optimization process. Such an approach is different from other popular architectures for asset health monitoring like LSTM networks and other supervised learning techniques. Our choice of architecture is mainly motivated by the lack of good quality labels in industrial use cases. Most of the assets are replaced before they fail, so it is not possible to determine precisely its remaining useful life. Moreover, asset breakdowns are rare events, therefore the number of run-to-failure cases in a real data set is usually limited. Autoencoders solve the problem as they do not use the labelled data for training—instead the model tries to learn a hidden representation of the process and by comparing the reconstruction with the original data, we are able to determine if the given observation is within the learned process boundaries. The variational autoencoder architecture is used to ensure that the observations are better structured in the latent space, which we believe may improve the prediction accuracy. The schematic diagram of our prediction model is shown in Figure 9. We have compared our solution with a baseline model, which is a deep autoencoder that computes its anomaly score using the mean squared error between input data and reconstruction.

We use our solution on the two described data sets. For both data sets we apply the same procedure. Before training the model, we split the data into three subsets: train, test and validation, each having 70%, 15%, 15% of all data, respectively. Based on the data from the training set, a linear scaling is performed to fit all features into 0–1 range and the same scaling is applied to the test and validation sets.

We have built our neural network model with the use of Tensorflow [39] and Keras [40] libraries in Python. To find the optimal architecture of the neural network, we have specified the hyperparamaters that we tried to optimize. The list of hyperparameters connected to the model architecture includes the number of neurons in the latent space, the number of fully connected layers in the encoder/decoder, the activation function after each layer and the dropout rate for regularization. The number of neurons in each layer was determined based on the shape of the input and latent space. The first layer in the encoder always has the same size as the input. Each consecutive layer is then a product of the number of neurons in the previous layer and the compression factor, which is calculated as shown in Equation (4). The number of neurons at *i*th layer of the encoder is determined by Equation (5).

(4) $$c={\left(\frac{n_{z}}{n_{0}}\right)}^{\frac{1}{d}}$$

where *c* is compression factor, $n_{z}$ is the size of the latent space, $n_{0}$ is the input size and *d* is the number of fully connected layers.

(5) $$n_{i}=n_{i-1}c$$

where $n_{i}$ is the number of neurons at *i*th layer, $n_{i-1}$ is the number of neurons at previous layer and *c* is the compression factor.

Shape of the decoder is the mirror image of the encoder shape. It means that the first layer of the decoder has the same number of inputs as the last layer of the encoder and so on. Determination of the number of neurons in such a way ensures that we have a unified compression at each layer of the neural network and limits the number of hyperparamters defining the shape of the model to only two variables (latent size and number of layers). We also optimize the value of the anomaly threshold that will be used as a boundary between normal and abnormal observations. We define a “quantile” hyperparameter, which is determining the threshold based on the chosen percentile of the train reconstruction error.

**Figure 10:**
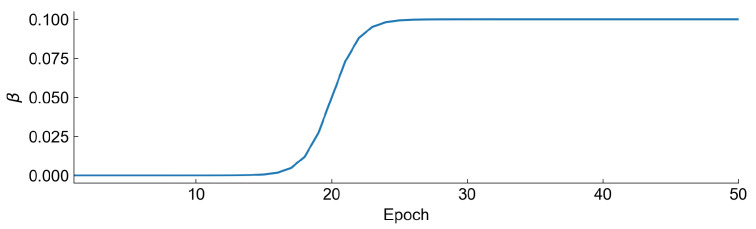
β annealing curve with $\beta_{max}=0.1$, $n_{max}=50,n_{0}=10$.

In the training process of the autoencoder, we have set two additional hyperparameters that were optimized—this is the number of epochs and batch size. To ensure low reconstruction loss of variational autoencoder, we have decided to use a β annealing technique in our study. We always start with β=0 and start to increase it at some epoch to a $\beta_{max}$ value like a sigmoid function. The value of beta at each epoch is defined as:

(6) $$\beta_{n}=\frac{\beta_{max}}{1+e^{-n+n_{max}-n_{0}}}$$

where $\beta_{n}$ is the value at *n*th epoch, $\beta_{max}$ is the maximum value, *n* is the number of epochs, $n_{max}$ is total number of epochs and $n_{0}$ is the hyperparameter defining when β starts to increase rapidly. Exemplary shape of the annealing curve is represented in Figure 10.

We then define a reconstruction loss to be a weighted sum of Kullback Leibler divergence and reconstruction loss (mean squared error between the input and output of the autoencoder):

(7) $$L=\beta L_{KL}+L_{x}$$

where *L* is total loss, $L_{KL}$ is Kullback-Leibler divergence loss and $L_{x}$ is the reconstruction loss.

As the cost function for the optimization, we have selected the F1 score, which is the harmonic mean of precision and recall. We have decided to use this metric for evaluating our model due to the fact of having very imbalanced data sets, where anomalies are only a fraction of all observations. The F1 score is calculated based on the test set. The optimization of the model is done with the use of hyperopt [41] library using Bayesian Optimization with the Tree-structured Parzen Estimator. Table 1 summarizes all hyperparameters used and their domains.

Due to the different number of hyperparameters, we optimize a “simple” autoencoder model with 100 evaluations and a variational autoencoder with 150 evaluations. We save the best result of each model and then validate its F1 score on the evaluation set, which is the final performance metric of the model.

At the end, we apply the SHAP method to find the most important features related to the classification of the observations as anomalies. To speed up the computation time of SHAP, instead of using a model-agnostic approach with Kernel Explainer, we create a surrogate model with Random Forest algorithm [42] and use Tree Explainer, which lowers the computational complexity from $O(TL2^{M})$ to $O(TLD^{2})$, where T is the number of trees, L is the maximum number of leaves, M is the number of features and D is the depth of a tree [43].

## 6. Experimental Results

### 6.1. C-MAPSS Data Set

In this section we present the results of our research on the C-MAPSS dataset. We first present the hyperparameters found by the optimization algorithm for both “simple” and variational autoencoders (Table 2). Then we present the detailed metrics of each model and show differences i.e., in the formed latent space of the autoencoders.

**Figure 11:**
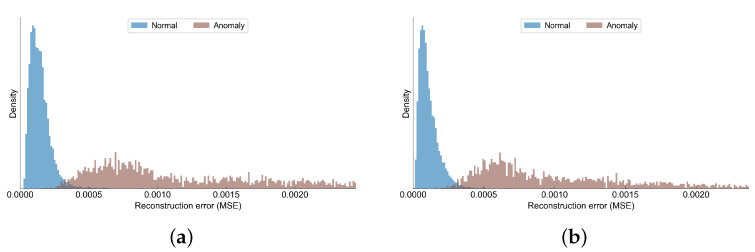
Reconstruction loss of normal and anomaly observations in the C-MAPSS data set. (**a**) AE; (**b**) VAE.

In the validation set, we have collected 10,242 observations, out of which 1575 were marked as anomalies. The confusion matrices in Table 3 show the detailed predictions of AE and VAE models. Table 4 presents a summary of the different metrics obtained by autoencoder and variational autoencoder. Although both models obtain very good metrics, the variational autoencoder slightly outperforms the simple autoencoder. In Figure 11 we have shown how the reconstruction errors of normal observations differ from the anomalous observations. Although there is some small overlapping between the distributions of the errors in each class, the anomalies tend to have much higher reconstruction errors compared to normal samples.

**Figure 12:**
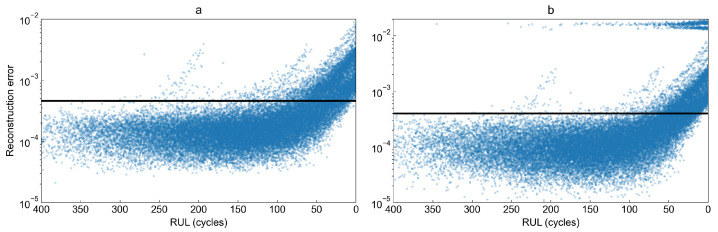
Reconstruction loss as a function of remaining useful life for (**a**) autoencoder and (**b**) variational autoencoder.

To observe how the reconstruction error behaves as the remaining useful life decreases, we have plotted the reconstruction error as a function of RUL for both studied models in Figure 12. Visual inspection of both plots shows very high similarity between them. However, the variational autoencoder for some observations computes an extremely high reconstruction error compared to other values. The figure shows that the first signs of degradation process may be observed when there are between 50 and 90 cycles left until failure.

**Figure 13:**
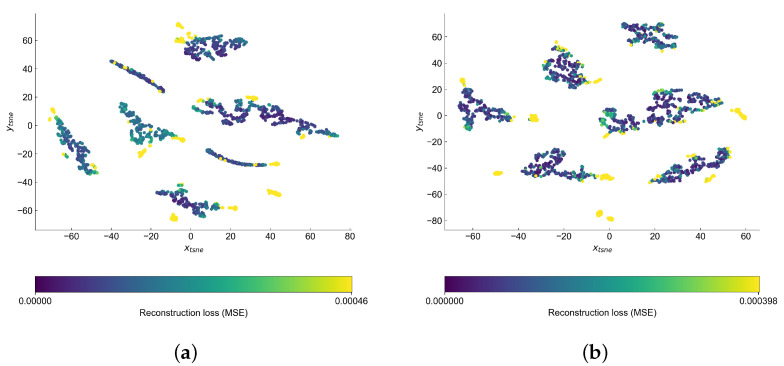
Latent space of the autoencoders in the C-MAPSS data set reduced to 2D with use of t-SNE. (**a**) AE; (**b**) VAE.

Visual inspection of the autoencoder’s latent space may help us better understand the structure of the data and find the reasons behind the points being marked as anomalies. This should be especially valid in the case of VAE, where there is extra attention paid to forming this space. As in both cases the latent space has higher dimension than two, we use t-SNE [44] implementation from sckit-learn [45] to reduce the latent space into two-dimensional space, which is easier for analysis by eye. In both cases, we set the perplexity hyperparameter to 20. The latent space of both models is presented in Figure 13.

Both latent spaces create clusters, which seem to be different operational conditions. The anomalies are generally on the edge or outside of the clusters—this is a bit easier to notice in the case of variational latent space. We observe the biggest difference in the shape of the clusters. In AE latent space the clusters are rather long and narrow, while in the case of VAE they try to form more of a circular cluster.

For the purpose of anomaly explanations, we have trained a Random Forest Classifier (with 16 estimators and rest parameters set as default in its sckit-learn implementation) on the evaluation data set. The inputs to the classifier were the scaled measurements and the output was the anomaly label predicted by VAE. As we only want our surrogate model to be able to reflect the validation data set and we do not need a generalization, the model’s accuracy is calculated also based on validation labels. The F1 score of the surrogate model is 99.8%, which means it makes almost identical predictions as our original VAE.

**Figure 14:**
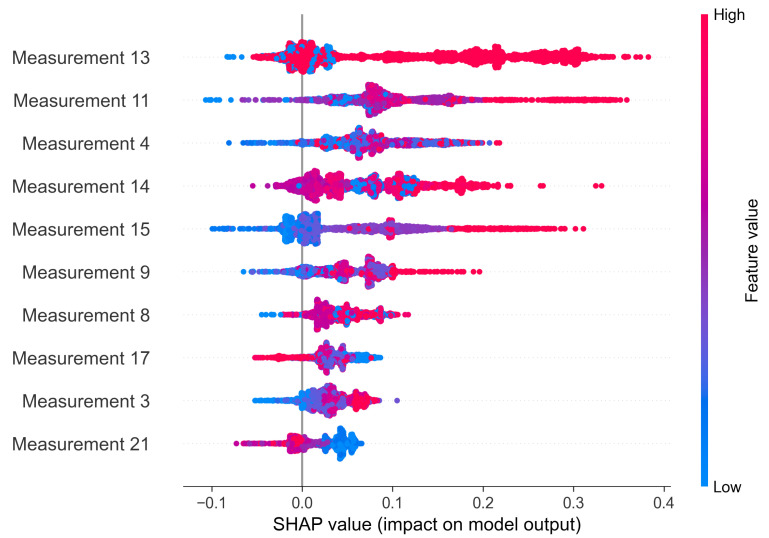
Global explanations of VAE model based on Shapley values—C-MAPSS data set.

The SHAP method provides both global and local explanations. Thanks to that, it is possible to draw a conclusion on the anomaly source of one sample and of the whole system. In Figure 14 we present a beeswarm plot summarizing the impact of each feature on the final anomaly. As the Shapley value increases, it means that a certain value of a feature favors the classification of the observation as anomaly. The features are sorted based on the mean Shapley value, therefore the features at the top of the plot are considered as the most important ones.

**Figure 15:**
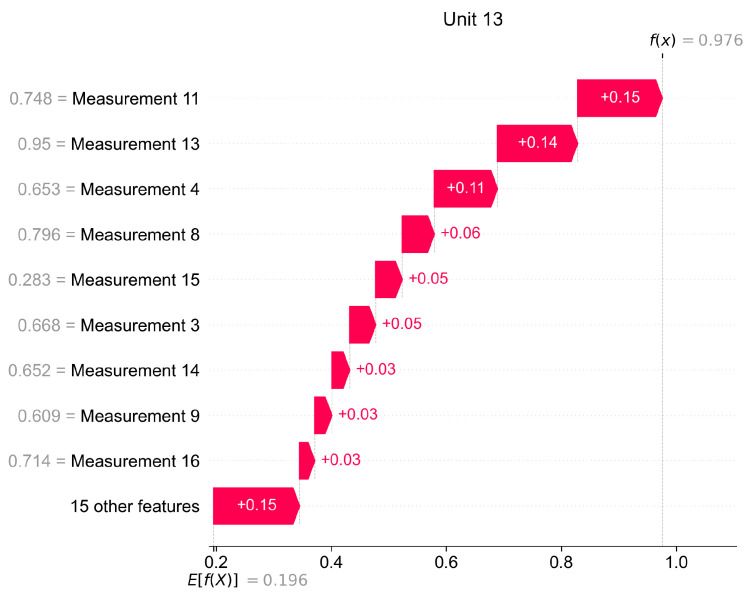
Local explanations of VAE model for a selected turbofan engine unit based on Shapley values.

In Figure 15 we present how Shapley values can be used for giving local explanations. The presented values are calculated as the mean of observations classified as anomaly, therefore this plot delivers information about the most important features which caused the anomaly in a given unit.

### 6.2. Hot Rolling Data Set

The procedure for evaluation of the hot rolling data set results is the same as in the case of C-MAPSS data set. We begin with a presentation of optimized hyperparameters for both models, which can be found in Table 5. Then we show the confusion matrices for the evaluation data set (Table 6) and score metrics (Table 7).

The results for the AE model show that the model has very high precision (almost 90%), this means that there is a relatively low number of false positives. The value of the recall metric is much lower, which means many of the anomalies are not properly labelled. The VAE model was able to correctly classify more anomalies and achieved a higher F1 score than AE model, however its precision is lower. This is balanced by the higher recall score than in the case of AE.

The overall performance of the model may be acceptable if the model was to be used only as an additional help for the operators and engineers. However, its indications must be considered with care. We believe that the relatively low performance may be due to one of two reasons: (1) we do not have run-to-failure data and therefore some of the products which were considered as anomalous (due to the assumption we have made regarding the length of rolling campaigns) are good working conditions, or (2) the model requires more fine-tuning and pre-processing, as it is not able to properly separate anomalies from normal observations.

**Figure 16:**
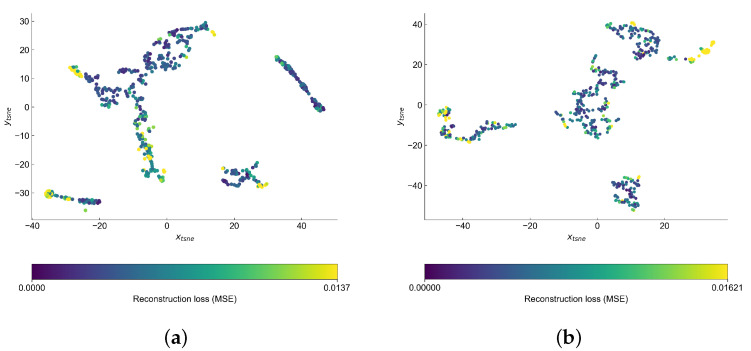
Latent space of the autoencoders in the HRM data set reduced to 2D with use of t-SNE. (**a**) AE; (**b**) VAE.

Inspection of the latent spaces (Figure 16) show that our data formed four clusters. The anomalies are often on the edges of clusters, although the anomalous points seem to partially overlap with the normal samples. The clusters are better separated in case of VAE model and we can observe that some of the anomalous points are found outside four main clusters.

**Figure 17:**
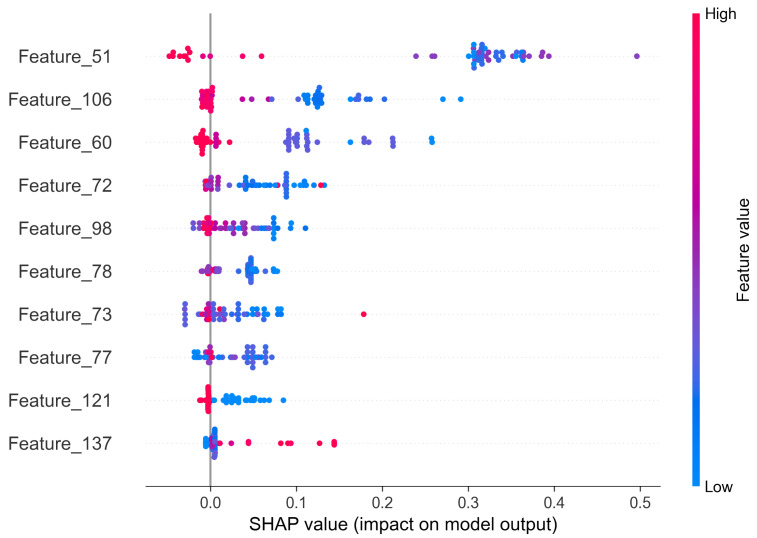
Global explanations of VAE model based on shapely values—HRM data set.

Usage of the SHAP method (in the same way as described in C-MAPSS data set) brings valuable information towards understanding the source of anomalies. In Figure 17 we can see that Feature No. 51 has definitely the greatest impact on the classification task. The general trend is that the values of the features decrease with the roll wear. However, what we must point out is that the surrogate model did not perform very well, as its F1 score was equal to 85%, which means that not all anomalies were properly assigned.

## 7. Discussion

In this paper we have discussed the topic of predictive maintenance in complex industrial systems such as hot strip mill. We have focused on the problems of implementing practical predictive maintenance solutions in the industry, from which the major one is the lack of well-labeled failure data. To overcome this issue, we have proposed to treat the degradation of an industrial asset as an unsupervised learning problem, where less labeled data is needed to obtain decent predictions. We proposed a deep learning algorithm which uses a variational autoencoder for anomaly detection in the observations. We have also investigated the problem of explainability of black-box models, which is another important obstacle to bring predictive maintenance from research to industry. We expect the developed solution to be helpful for health monitoring of work rolls, which is a key asset in a hot strip mill, determining the length of rolling campaigns and having a significant impact on the quality of the produced coils. Currently, the rolls are replaced after rolling a predefined length of steel. Switching the strategy to intelligent monitoring of work rolls may help to take better decisions during the planning and production phases—for example, by extending the rolling campaigns where possible. The choice of an unsupervised learning model was driven by the data availability in real-life applications. We rarely have enough data from run-to-failure situations, which allow us to determine the remaining useful life and efficiently train supervised learning algorithms such as LSTM networks. Making use of autoencoder-based architecture removes the above problem by using only data from normal operation conditions in the training phase. Although our work is focused on a hot strip mill, we also looked in detail at C-MAPSS data set, which is state-of-the-art data for developing data-driven PdM solutions and can be easily evaluated with the work of other researchers. The study has shown that variational autoencoders have a possibility to outperform the traditional autoencoder architecture, as the results we obtained are promising, and in our study VAE has slightly outperformed the AE architecture. The results we obtained in the case of hot rolling mill data are significantly worse than the one we observed in the previous data set. We believe that the lower metrics on this data set may be coming from the fact that this is a real use case and the variance within the process is much higher than on simulated data, which is much more repetitive.

Nevertheless, we believe that the detection of anomalies with such accuracy might be helpful for the operators and engineers in the development phase of predictive maintenance solutions. Although it is required to increase the performance of such models to raise trust in the predictions of machine learning models. The improvement of the model might require the acquisition of new data that at this moment might not be in the data set or some more advanced data preprocessing.

Comparing both implemented models, the differences in metrics were (just like with the C-MAPSS data set) not significant, however VAE again turned out to be a bit more accurate. The use of the SHAP method for explanation of anomalies has also shown potential, as it was able to correctly indicate the measurements, which were changing as degradation progressed. Such information may be useful to the system operators or maintenance engineers and could ease the process of failure identification. As our final model might be considered a binary classifier, it is not capable of distinguishing between different types of failures. Successful implementation of such a failure classifier could definitely bring great value to the industry, however obstacles remain which justify the development of simpler models. Nevertheless, we plan to take different approaches towards the development of predictive maintenance models to bring industry-ready solutions.

## Figures and Tables

**Table 1:** List of hyperparameters used in the models.

Name	Domain	Value Range
*All models*		
Layers	integer	2–4
Latent size	integer	2–10
Activation	choice	relu, elu, tanh
Dropout	real	0.0–0.3
Batch size	choice	32, 64
Epochs	integer	30–50
Quantile threshold	real	0.9–1.0
*Variational autoencoder*		
$\beta_{max}$	real	0.001–0.1
$n_{0}$	integer	3–30

**Table 2:** The obtained hyperparameters from the C-MAPSS data set.

Hyperparameter	AE	VAE
Layers	4	3
Latent size	6	4
Activation	elu	elu
Dropout	0.03	0.16
Batch size	64	64
Epochs	32	39
Quantile threshold	0.99	992
Beta max		0.001
Epochs Beta		28

**Table 3:** Confusion matrices for C-MAPSS data set.

		AE		VAE	
		**Predicted**			
		Normal	Anomaly	Normal	Anomaly
**Actual**	Normal	14,406	150	14,443	113
	Anomaly	172	2453	111	2514

**Table 4:** Metrics summary for HRM data set.

Metric	AE	VAE
Accuracy	98.1%	98.7%
Precision	94.2%	95.7%
Recall	93.4%	95.6%
F1	93.8%	95.7%

**Table 5:** The obtained hyperparameters form HRM data set.

Hyperparameter	AE	VAE
Layers	3	4
Latent size	7	10
Activation	relu	relu
Dropout	0.102	0.115
Batch size	32	32
Epochs	45	34
Quantile threshold	0.930	0.916
Beta max		0.0081
Epochs Beta		8

**Table 6:** Confusion matriices for HRM data set.

		AE		VAE	
		**Predicted**			
		Normal	Anomaly	Normal	Anomaly
**Actual**	Normal	435	6	431	10
	Anomaly	45	41	40	45

**Table 7:** Metrics summary for C-MAPSS data set.

Metric	AE	VAE
Accuracy	90.5%	90.5%
Precision	87.2%	81.8%
Recall	48.2%	52.9%
F1	62.1%	64.3%

## Data Availability

*C-MAPSS*: Publicly available datasets were analyzed in this study. This data can be found here (accessed 30 November 2021): https://ti.arc.nasa.gov/c/6/. *Hot rolling*: Restrictions apply to the availability of this data. Data was obtained from ArcelorMittal Poland and is not publicly available due to confidentiality issues.
